# Efficacy of Smartphone Apps in Patients With Depressive Disorders: A Systematic Review

**DOI:** 10.3389/fpsyt.2022.871966

**Published:** 2022-08-12

**Authors:** Alba Hernández-Gómez, María José Valdés-Florido, Guillermo Lahera, Nelson Andrade-González

**Affiliations:** ^1^Department of Personality, Assessment and Clinical Psychology, Complutense University of Madrid, Madrid, Spain; ^2^Psychiatry Service, University Hospital of Gran Canaria Dr. Negrín, Las Palmas de Gran Canaria, Spain; ^3^Faculty of Medicine and Health Sciences, University of Alcalá, Alcalá de Henares, Spain; ^4^IRyCIS, CIBERSAM, Madrid, Spain; ^5^Príncipe de Asturias University Hospital, Alcalá de Henares, Spain; ^6^Psychiatry and Mental Health Research Group, Faculty of Medicine and Health Sciences, University of Alcalá, Alcalá de Henares, Spain; ^7^Faculty of Medicine, Alfonso X el Sabio University, Villanueva de la Cañada, Madrid, Spain

**Keywords:** depression, smartphone, efficacy, app, systematic review

## Abstract

**Background:**

Digital interventions have become an accessible format in clinical practice to provide better support for patients with mental disorders. However, the clinical efficacy in patients with depressive disorders is not well known. We aimed to determine the efficacy of smartphone applications (apps) in patients diagnosed with a depressive disorder.

**Method:**

An electronic database search was performed of PubMed, PsycINFO, and Web of Science, to identify relevant articles up to June 12, 2021. Peer-reviewed articles were screened and selected based on predetermined inclusion and exclusion criteria.

**Results:**

Seven articles met the inclusion criteria and therefore were selected for the systematic review, which included a total of 651 patients. The results were heterogeneous, essentially due to the different methodologies used in the selected studies.

**Conclusions:**

Digital smartphone-delivered interventions do not appear to reduce depressive symptomatology nor improve the quality of life in patients diagnosed with depressive disorders when compared to an active control group. Taking into account the inherent methodological difficulties and the variability among such studies, it is apparent that further research—with more methodologically refined clinical trials, including larger sample sizes—is needed.

## Introduction

Major depressive disorder (MDD) is a mood disorder characterized by low or depressed mood present for two weeks or more, along with other symptoms such as loss of interest or pleasure, loss of energy, sleep and appetite disorders, diminished ability to concentrate and recurrent negative thoughts as main features (1). The lifetime prevalence of MDD varies from 2 to 21% (2), presenting approximately twice as often in women than in men (3). MDD is one of the medical conditions that generates the largest chronic disease burden adjusted by “years lived with disability” in populations of any socioeconomic status. Additionally, MDD increases the risk of developing other medical conditions [i.e., cardiovascular and neoplastic diseases, (3)]. Social and health care costs associated with MDD are high (4), and patients still have barriers to access to quality treatment. Since the emergence of the COVID-19 pandemic, the prevalence of depression and anxiety has continued to increase globally (5). This points to the need for urgent and cost-efficient solutions for people experiencing this symptomatology.

The progress of new technologies is having an impact on clinical practice in mental health. In regard to developing improvements in clinical attention for people with mental health disorders, smartphone apps are in the spotlight. App interventions delivered via smartphones are low cost and of special relevance for public health (6). Apps are flexible interventions that can be therapist-guided, unguided [i.e., self-help apps; (7)], or applied as a stand-alone or combined treatment.

Regarding psychotic disorders, the literature suggests that app interventions are feasible and well accepted by people with such disorders, especially during early stages of the disease (8), and therefore may improve treatment adherence, symptomatology, and duration of hospital admissions (9). In addition, evidence suggests that people presenting with more severe psychotic and depressive symptoms seem to have a better engagement with digital interventions compared to people experiencing mild symptoms (10). In this vein, apps seem to improve manic and depressive symptomatology in people with bipolar disorder, especially when interventions include psychoeducation techniques (11) without any apparent limitation in their use by these patients (12). On the other hand, to date, possible negative effects associated with the use of digital interventions in patients with depressive disorders have been detected, such as the appearance of new symptomatology, worsening of the existing disorder, trouble when implementing the designated tasks or a subjective feeling of pressure while trying to complete them on time (13). Similarly, other barriers, such as worry about the efficacy of the intervention, difficulties in app use, privacy issues and lack of direct professional feedback, were detected (14).

Regarding anxiety and depressive disorders in particular, the literature suggests that apps may have a role in reducing anxious-depressive symptomatology. One systematic meta-review of meta-analyses revealed that the use of mobile-based interventions is a promising approach to achieving a modest reduction in depressive and anxious symptoms, although there are no clear differences when such interventions are compared to active therapeutic interventions (6). In terms of assessing the extent of the clinical improvement, it is paramount to consider the level of intervention adherence, which is greater when the intervention involves a higher degree of user engagement (15). However, maintaining user engagement is a challenge in relation to app interventions because, often, there is a rapid drop in their use (6). In a previous systematic review, contradictory results regarding the efficacy of apps based on Cognitive Behavioral Therapy (CBT) in patients suffering from mild and moderate depression were found (16). According to Hrynyschyn and Dockweiler (16), the clinical and methodological heterogeneity in the clinical trials interferes with the outcome analyses and prevents us from drawing solid conclusions. However, the review conducted by Hrynyschyn and Dockweiler (16) provides limited evidence about the efficacy of these interventions, since it focuses exclusively on apps based on CBT, includes patients with mild or moderate depression selected by using cutoff scores in questionnaires about depression (without the need of being diagnosed by a health professional) and only analyzes studies carried out from 2015 onward. In their meta-analysis, Serrano-Ripoll et al. (17) found smartphone app interventions had a moderate effect on depressive symptomatology. Nonetheless, these authors include studies conducted with participants with depressive symptoms, and not in all the studies were the patients diagnosed by a health professional. Additionally, in this systematic review, only studies that compared app interventions to non-active control groups (i.e., minimal intervention, treatment as usual, waiting-list control) were analyzed. This could overestimate the effect of app interventions.

Some studies have highlighted the advantages of digital interventions, such as the motivational aspects of the apps and their accessibility (18). This is especially relevant to those individuals for whom it is more difficult to access face-to-face clinical appointments (19). Despite these advantages, implementing digital interventions still presents numerous challenges before such interventions can be incorporated into daily clinical practice, such as the lack of integration of apps into computer systems in public health care, or issues surrounding the protection of client privacy (20). This contrasts with the increasing worldwide popularity of apps among smartphone users, which is leading to an overload of the app market (21), along with the proliferation of apps that employ non-evidence-based techniques (22). In line with the aforementioned and as a result of the mental health problems associated with the COVID-19 pandemic, the rise of apps continues to increase (23). Thus, a systematic review to critically analyze and reflect on the efficacy of such apps is needed.

Given the high availability of these apps in app stores (21) and their growth in both the app market and clinical practice (20), it is necessary to critically assess the efficacy of these interventions in patients with depressive disorders diagnosed by a health professional. Therefore, the aim of the present systematic review is to determine the efficacy of smartphone app interventions for patients with depressive disorders.

## Methods

To achieve the aims of the present systematic review, the recommendations of the Preferred Reporting Items for Systematic Reviews and Meta-Analyses (PRISMA) Statement (24) were followed.

### Study Selection Criteria

The inclusion criteria were as follows: (a) randomized clinical trials regarding the efficacy of apps (based on different theoretical orientations, not only CBT) in patients with a diagnosis of depression, including another intervention (active control group) as a comparative group^1^; (b) studies written in English and published in peer-reviewed indexed journals up to June 12, 2021; (c) studies conducted in adult and/or adolescent populations with a primary diagnosis of MDD or another depressive disorder, with any clinical severity, reported by a health professional in accordance with the current psychiatric classifications of the ICD (International Classification of Diseases) or DSM (Diagnostic and Statistical Manual of Mental Disorders), in any edition^2^; and (d) use of a smartphone-delivered digital intervention. The exclusion criteria were as follows: (a) studies that were not a clinical trial, such as narrative or systematic reviews, meta-analyses, protocols, case series, congress conferences, and short communications, (b) studies carried out in populations without a diagnosis of mental disorder (or a diagnosis not reported by a health professional) or analog study, (c) studies including patients with depression and other clinical comorbidities (unless the primary diagnosis was a depressive disorder), (d) studies that did not include a smartphone app (i.e., computers, website intervention) as an intervention group. No temporal filter was used; thus, all evidence to date was screened up to June 12, 2021. Ethical approval was not required since this was a systematic review of published studies.

### Search Strategy

The PubMed, PsycINFO, and Web of Science databases were searched until June 12, 2021 to conduct this systematic review. The search strategy employed in these databases was (“Depression” OR “Depressive”) AND (“App” OR “Smartphone”). To meet the inclusion criteria, different filters were applied to each of the three databases (filters are available upon request from the corresponding author). Additionally, an informal manual search through Google Scholar was performed to identify possible articles of interest.

### Study Selection Process

The study selection was conducted in four phases. First, in the identification phase, articles were identified from the three databases, and duplicates were removed. Second, in the screening phase, the titles and abstracts of articles meeting the inclusion criteria were reviewed. When there were disagreements in the screening process, full texts were assessed independently by AH-G, MJV-F, NA-G, and GL. Finally, in the eligibility and inclusion phases, articles were examined entirely and finally selected for inclusion in the systematic review.

### Data Extraction Process

The following information was extracted from the selected articles: (1) authors and year of publication, (2) sample characteristics (average age, size, diagnoses, diagnostic method employed), (3) treatment type in the intervention group (number and components used, therapeutic orientation), (4) treatment type in the control group (active control group, treatment as usual), (5) outcome measures and methods of assessment, (6) number of assessments and follow-up, and (7) key findings. These data were coded independently by the authors and included in a previously standardized designed template to register homogeneously all the information for the analysis, synthesis, and interpretation of the results. Any disagreement in the data recorded was resolved through a reasoned discussion until a consensus was reached.

## Results

**Figure 1:**
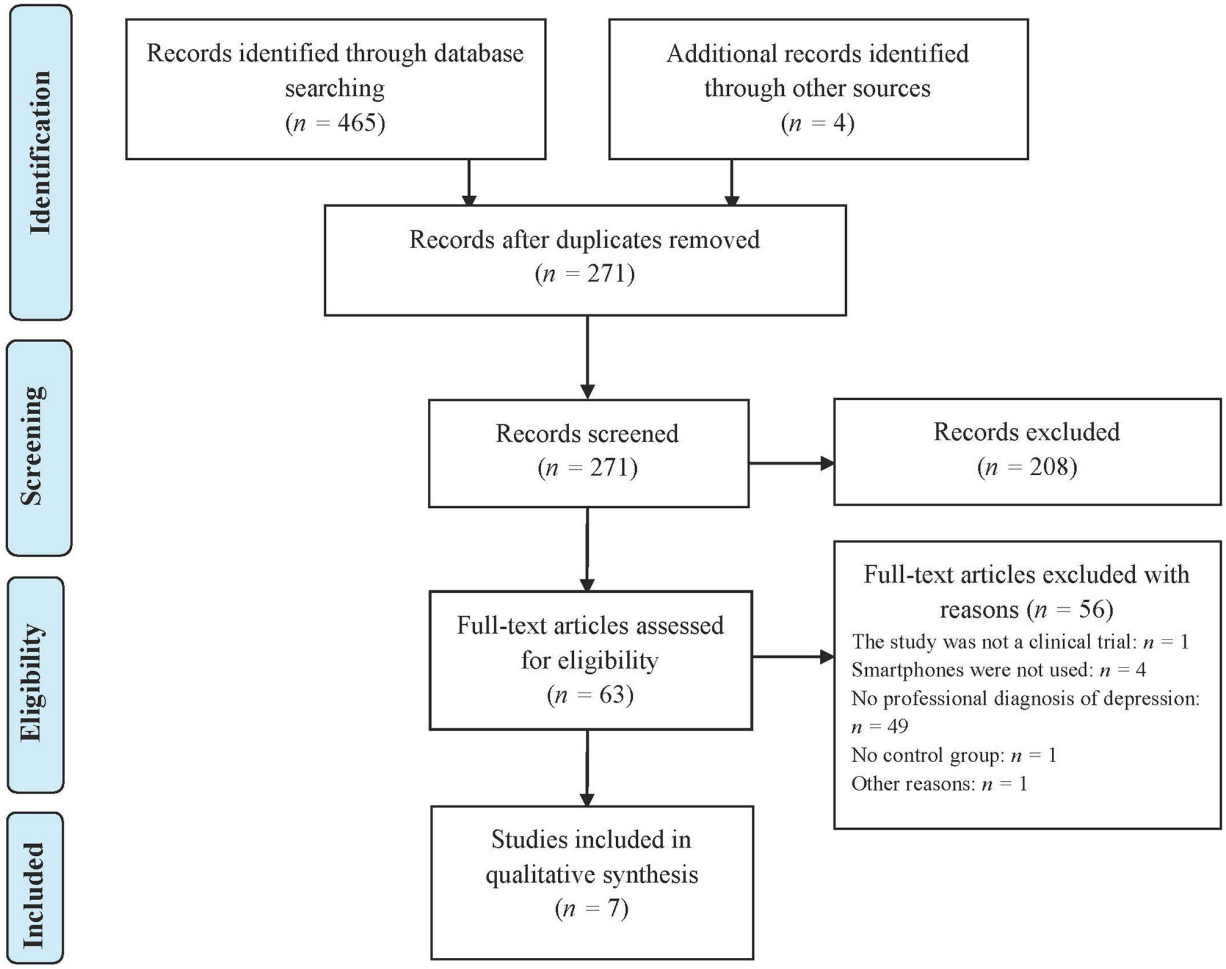
PRISMA flow diagram of the article selection process.

Seven studies that met the inclusion criteria were selected. Figure 1 shows the selection process of the articles included in this systematic review. The main characteristics of the selected studies are shown in Table 1.

**Table 1:** Main characteristics of the selected studies included in this systematic review to examine the efficacy of app interventions in patients with depressive disorders.

**Author**	**Sample**	**Type of intervention in the** **study group**	**Type of intervention in** **the control group**	**Outcome measures and** **methods of assessment**	**Number of measurements** **and follow-up**	**Key findings**
Hur et al. (25)	M_age_ = 23.71 years. 88.24% females. Participants with other specified depressive disorder diagnosis (according to DSM-5), with BDI-II score ≥ 10. Diagnostic method: SCID-NP. *n =* 34	TT app based on CBT and *ad hoc* questionnaire about possible situations in life scenarios, tasks three times a day. Components: (1) Identifying cognitive distortions after reading a short story. (2) “Decatastrophizing questions”: diverse scenarios and realistic and unrealistic options to choose. (3) “Distancing questions”: asking the participants to imagine what the other person would do in the displayed situation. Human support: none.	Daily use of an app (10–15 min per day) recording mood state, and sleep quantity and quality.	Dysfunctional attitudes (DAS), depressive symptoms (BDI-II), anxiety (STAI-X2), quality of life (QOLI), self-esteem (RSES).	Two measurements (at baseline, and at 3 week after the app usage).	Significant improvement in dysfunctional attitudes and anxiety after 3 weeks of using the app in the intervention group. No significant differences in depressive symptoms, self-esteem, and quality of life between groups.
Ly et al. (26)	M_age_ = 36.1 years. 70% females. Participants with MDD diagnosis (according to DSM-IV), with at least an episode in partial remission. Diagnostic method: MINI. Total score ≥5 on the PHQ-9 scale. *n =* 81	App based on BA. Components: (1) Psychoeducation about BA. (2) BA (participants could register their behaviors to increase their activation). Human support: minimal therapist contact (maximum of 20 min per participant and week). Participants received short encouraging messages and general educational messages from their therapist; and participants received personal feedback about the activities they had performed from the therapist *via* email.	App based on mindfulness. Components: (1) Psychoeducation about mindfulness. (2) Audio tracks with exercises (both guided and unguided, 3 min or 30 min) for mindfulness practice. Human support: minimal therapist contact. Mails sent by the therapist (maximum of 20 min per participant and week). Participants received short encouraging messages and general educational messages from their therapist *via* email. Participants received therapist feedback about a weekly reflection.	Primary outcome measures: depressive symptomatology (BDI-II and PHQ-9). Secondary outcome measures: quality of life (QOLI), anxiety (BAI), and experiential avoidance (AQQ-II). Recovery: the diagnostic criteria for depression are no longer fulfilled according to the diagnostic interview used.	Primary outcome measures: pretreatment, post-treatment, 6-month follow-up measures. Three measurements of the BDI-II. The PHQ-9 was applied on a weekly basis during 8 weeks (10 measurements). Secondary outcome measures: pretreatment, post-treatment, 6-month measure after the start of the treatment. Three measurements of the BAI and QOLI. AQQ-II applied on a weekly basis for 8 weeks (10 measurements).	No significant differences in depressive symptoms between groups, either at post-treatment (*d* = 0.25 for BDI-II; *d* = 0.28 for PHQ-9) or at the 6-month follow-up (*d* = 0.03 for BDI-II; *d* = 0.15 for PHQ-9). No significant differences in anxiety, experiential avoidance, quality of life, and recovery rates between groups, either at post-treatment or at the 6-month follow-up.
Ly et al. (27)	M_age_ = 30.60 years. 69.9% females. Participants with MDD diagnosis (according to DSM-IV). Diagnostic method: MINI. Total score ≥5 on the PHQ-9 scale. *n =* 93	Blended treatment: App based on BA and four face-to-face sessions based on BA. Components: (1) Psychoeducation. Follow-up on homework: modify activity plan and homework assignment. (2) Participants could register their non-depressed behavior, with the possibility of adding a personal reflection. (3) Set relapse prevention plan. Human support: encouraging messages every 2–3 days, and weekly educational messages sent by the therapist.	Full BA: ten face-to-face sessions of BA, with homework and activity schedules for the participant to complete in the time between the sessions. Components: (1) Psychoeducation. (2) Homework assignment and follow-up. (3) Discussion about relapse prevention, and set relapse prevention plan.	Primary outcome measures: depression symptoms (BDI-II). Secondary outcome measures: depression symptoms (PHQ-9), quality of life (QOLI), anxiety (BAI), and experiential avoidance (AQQ-II). Recovery: participants were considered recovered when they improved ten or more points in the BDI-II.	Primary and secondary outcome measures except PHQ-9: pretreatment, post-treatment, follow-up (six months after the end of the treatment) (3 measurements). PHQ-9 applied on a weekly basis (13 measurements).	No significant differences in depression between the intervention and control groups, either at post-treatment (*d* = −0.13 for BDI-II; *d* = 0.01 for PHQ-9) or at the 6-month follow-up (*d* = −0.10 for BDI-II; *d* = 0.05 for PHQ-9). No significant differences in quality of life, anxiety, experiential avoidance, and recovery rates between the intervention and control groups, either at post-treatment or at the 6-month follow-up.
Mantani et al. (28)	M_age_ = 40.90 years. 53.5% females. Participants with MDD diagnosis without psychotic features (according to DSM-5) and antidepressant resistance (BDI-II score ≥ 10). Diagnostic method: Primary Care Evaluation of Mental Disorders procedure. *n =* 164	Kokoro-App (eight 20-min sessions based on CBT) + switching their antidepressant either to escitalopram (5–10 mg/day) or to sertraline (25-100 mg/day). Components: (1) One welcome session. (2) Two sessions on self-monitoring. (3) Two sessions on BA. (4) Two sessions on cognitive restructuring. (5) Epilog focusing on relapse prevention. (6) Psychoeducation. Human support: participants received a personalized congratulatory email for their progress. Face-to-face CBT or interpersonal therapy was prohibited during 9 weeks.	Only switch their previous antidepressant to escitalopram or sertraline. Human support: face-to-face CBT or interpersonal therapy was prohibited during 9 weeks.	Depression symptoms (PHQ-9, BDI-II) and frequency, intensity, and burden of side effects (FIBSER).	Five measurements (at 0, 1, 5, 9 and follow-up at 17 weeks).	Intention-to-treat analysis for the total sample: significant improvement in depression symptoms at 9 weeks in Kokoro-App group (OR = −2.48 for PHQ-9; OR = −4.1 for BDI-II). Per-Protocol Sample (i.e., participants' adherence to medication, with mild or fewer side effects than at baseline and still symptomatic, *n* = 117): significant improvement in depression symptoms (PHQ-9, not BDI-II) at 9 weeks and fewer side effects in Kokoro-App group. Participants in the intervention group maintained their improvement for 8 weeks. No statistically significant differences in burden of side effects (total sample). When participants of the control group accessed the smartphone app, both the app intervention group and the control group had similar results in depression symptoms and burden of side effects at week 17, for both the total sample and the per-protocol sample.
Raevuori et al. (29)	M_age_ = 25.1 years. 72.6% females. Participants with MDD diagnosis (according to ICD-10). Diagnostic method: diagnosis of a major depressive disorder (single or recurrent episode) documented in the medical records. *n =* 124	Therapist-guided intervention *via* a smartphone app. Components: combination of components of CBT, mindfulness-based stress reduction, mindfulness-based cognitive therapy, and BA. Eight weekly modules (daily practice: 10–45 min) that included text, videos, audio, infographics, and journal prompts (psychoeducation). Human support: moderated group discussion board and asynchronous support by a remote therapist that reviewed engagement and supported the participant *via* chat or phone calls (in cases of clinical need).	TAU from Finnish health care system. Components: TAU could, or not, include antidepressant medication, laboratory tests, and appointments with healthcare professionals.	Depression symptoms (PHQ-9), anxiety (GAD-7), sleep disturbances (ISI), quality of life (EUROHIS-QOL-8), internalization of mindfulness skills (FFMQ-SF), perceived stress (PSS-10), resilience (Resilience Scale).	Five measurements (at 0, 4, 8, 20 and 32 weeks).	Significant improvement in internalization of mindfulness skills (at 20 and 32 weeks), perceived stress (at 20 and 32 weeks), and resilience (at 32 weeks) in app intervention group compared to the control group. No significant differences between app intervention group and control group in depression symptoms (*d* = 0.19 at week 4 for PHQ-9; *d* = 0.10 at week 8 for PHQ-9; *d* = −0.16 at week 20 for PHQ-9; *d* = −0.32 at week 32 for PHQ-9), anxiety, sleep disturbances, and quality of life.
Tønning et al. (30)	M_age_ = 43.95 years. 52.45% females. Participants with MDD diagnosis (according to ICD-10), with moderate-severe depression. Diagnostic method: SCAN. *n =* 120	App based on CBT, with a 2–5 min/day self-rating. Components: (1) Daily symptom monitoring and clinical feedback. (2) Psychoeducation (delivered as text and cartoons, to detect signs of relapse). (3) Cognitive restructuring. (4) Rumination-focused CBT. Human support: study nurses overviewed data three times a week and provided feedback. Participants received daily reminders to complete the self-rating questions. In cases of non-response for several days, nurses contacted the participant.	Danish standard treatment (with smartphone app installed to collect objective data, but without access to content).	Primary outcomes: rate and accumulated duration of psychiatric readmissions. Secondary outcomes: severity of depressive symptoms (HDRS-17), psychosocial functioning (FAST), and number of depressive episodes. Tertiary outcomes: perceived stress (PSS), quality of life (WHO-QOL-BREF), self-rated depressive symptoms (HAM-D6, BDI), recovery (RAS), empowerment (Roger's Empowerment Scale), adherence to medication (MARS), wellbeing (WHO-5), rumination (RRS), worrying (PSWQ), satisfaction with care (VSS-A), and BA (BADS).	Three measurements (at 0, 3 and 6 months).	Significant improvement in recovery in app intervention compared to control group. No significant differences between the app intervention group and control group in rates of readmissions, accumulated duration of psychiatric readmissions, depressive symptoms, psychosocial functioning, number of depressive episodes, quality of life, wellbeing, satisfaction, perceived stress, satisfaction with care, and BA.
Watts et al. (31)	M_age_ = 41.00 years. 80% females. Participants with MDD diagnosis (according to DSM-IV), and PHQ-9 scores of depression. Diagnostic method: MINI. *n =* 35	Smartphone version of “Get Happy Program”, based on CBT. Components: six lessons conducted over 8 weeks, following the story of a character with depression. Homework activities after each lesson and additional resources (i.e., assertiveness skills, sleep hygiene measures). Human support: clinicians send emails or make phone calls to participants until lesson 2. No more therapist contact was programmed, only in cases of clinical need (if the participant had deterioration in K-10 or if the participant initiated the contact).	Computer version of “Get Happy Program”, based on CBT.	Depressive symptoms (PHQ-9), nonspecific psychological distress (K-10), severity of depression (BDI-II).	Four measurements for PHQ-9 and K-10 (baseline, mid-treatment, post-treatment, and at 3-month follow- up). Three measurements for BDI-II (baseline, post-treatment, and 3-month follow-up).	No significant differences were observed in depressive symptomatology (*d* = −0.47 for PHQ-9; *d* = −0.37 for BDI-II), severity of depression, and psychological distress in the smartphone app intervention group compared to the control group.

*AQQ-II, Acceptance and Action Questionnaire; BA, Behavioral Activation; BADS, Behavioral Activation for Depression Scale; BAI, Beck Anxiety Inventory; BDI, Beck Depression Inventory; BDI-II, Beck Depression Inventory-II; CBT, Cognitive Behavioral Therapy; d, Cohen's d; DAS, Dysfunctional Attitude Scale; DSM-IV, Diagnostic and Statistical Manual of Mental Disorders (fourth edition); DSM-5, Diagnostic and Statistical Manual of Mental Disorders (fifth edition); EUROHIS-QOL-8, EUROHIS Quality of Life; FAST, Functional Assessment Short Test; FFMQ-SF, Five Facet Mindfulness Questionnaire Short Form; FIBSER, Frequency, Intensity and Burden of Side Effects Rating; GAD-7, Generalized Anxiety Disorder 7-item; HAM-D6, Hamilton Depression Scale 6 items; HDRS-17, Hamilton Depression Rating Scale 17 items; ICD-10, International Classification of Diseases (tenth revision); ISI, Insomnia Severity Index; K-10, Kessler 10-item Psychological Distress Scale; Kokoro, means “heart, mind, spirit” in Japanese; MARS, Medicine Adherence Rating Scale; MDD, Major Depressive Disorder; MINI, Mini-International Neuropsychiatric Interview; OR, Odds ratios; PHQ-9, Patient-Health Questionnaire 9; PSS, Cohen's Perceived Stress Scale; PSS-10, Perceived Stress Scale; PSWQ, Penn State Worry Questionnaire; QOLI, Quality of Life Inventory; RAS, Recovery Assessment Scale; RRS, Ruminative Response Scale; RSES, Rosenberg Self-Esteem Scale; SCAN, Schedules for Clinical Assessments in Neuropsychiatry; SCID-NP, Structured Clinical Interview for DSM-IV, Non-Patient edition; STAI-X2, State-Trait Anxiety Inventory; TAU, Treatment as Usual; TT, mind-growth program Todac Todac; VSS-A, Verona Satisfaction Scale-Affective Disorder; WHO-5, The World Health Organization-Five Wellbeing Index; WHO-QOL-BREF, WHO Quality of Life-BREF*.

The seven studies included a total of 651 patients. All of the participants were adults, and their weighted average age was 35.49 years. In six of the selected articles, the patients had a diagnosis of MDD (according to the ICD-10, DSM-IV or DSM-5), whereas in one of the articles, the diagnosis was other specified depressive disorder [according to the DSM-5; (25)]. The average percentage of females was 69.52%.

### Type of Intervention, Components, and Control Group

Five of the seven studies were based on elements of CBT [i.e., cognitive restructuring and psychoeducation; (25, 28–31)]. One of these aforementioned articles included components of CBT in conjunction with third-generation therapies, such as Mindfulness-Based Stress Reduction (MBSR), Mindfulness-Based Cognitive Therapy (MBCT), and Behavioral Activation Therapy [BA; (29)]. Two of the seven studies included BA in the app intervention (26, 27). Five of the interventions explicitly included psychoeducation components (26–30), whereas four incorporated some kind of self-registration (26–28, 30). Six of the seven articles selected applied interventions that allowed some degree of asynchronous support by a therapist, often on an exceptional basis (26–31). Last, Mantani et al. (28) included a CBT-based smartphone app in combination with an antidepressant switch.

Regarding control groups, the intervention was face-to-face in one of the seven studies included (27). On the other hand, two of the seven studies used smartphone-delivered digital interventions as a control group; one of them consisted of daily monitoring of mood and sleep quality and quantity (25), and the other included mindfulness techniques (26). Additionally, Tønning et al. (30) employed a standard treatment in combination with a smartphone app (which only allowed for the collection of objective data, with no access to content) as a control group. Watts et al. (31) used the computer-delivered “Get Happy Program”, while Mantani et al. (28) applied an antidepressant medication switch in the control group. In two of the selected studies, the app intervention was compared to the treatment as usual (TAU) in the pertinent country (29, 30).

### Outcome Measures

The main outcome variable in six of the seven studies was depressive symptomatology, as assessed through self-administered questionnaires such as the Patient Health Questionnaire [PHQ-9; (26, 28, 29, 31)] and the Beck Depression Inventory-II [BDI-II; (25–28, 31)]^3^. Tønning et al. (30) included the rate and accumulated duration of psychiatric admissions as the main outcome, whereas depressive symptoms through both the Beck Depression Inventory (BDI) and the Hamilton Depression Scale 6 items (HAM-D6) were assessed as secondary outcomes. Secondary and tertiary outcome variables were diverse, including assessments of quality of life (25–27, 29, 30), perceived psychological stress (29, 30) and unspecified psychological distress (31), anxiety levels (25–27, 29), medication side effects (28), internalization of mindfulness skills and resilience (29), dysfunctional attitudes (25), sleep disturbances (29), experiential avoidance (26, 27), self-esteem (25), adherence to medication (30), wellbeing (30), rumination (30), worrying (30), satisfaction with care (30), recovery (26, 27, 30), empowerment (30), psychosocial functioning (30), BA (30) and severity of depressive symptoms (30, 31).

### Number of Assessments

Regarding the number of assessments over time, the results were variable. Most studies made three measurements over time (26, 27, 30, 31). Hur et al. (25) included two measurements (at baseline and three weeks after using the app), whereas Ly et al. (26) took ten measurements of depressive symptomatology assessed by the PHQ-9 and ten measurements of experiential avoidance assessed by the AQQ-II. In the study by Ly et al. (27) depressive symptomatology was measured by means of the PHQ-9 thirteen times. Watts et al. (31) recorded four measurements of depressive symptomatology as assessed by the PHQ-9 and four indicators of nonspecific psychological distress, as assessed by the K-10 measure. Both Mantani et al. (28) and Raevuori et al. (29) recorded five measurements of their outcome variables. Despite the difference in the timing of the measurements, six studies incorporated at least one follow-up measurement (26–31).

### Efficacy on Depressive Symptomatology, Symptom Severity, Recovery, and Readmissions

Only Mantani et al. (28) found that patients who used an app on their smartphones had a greater improvement in their depressive symptomatology compared to the control group. However, six studies found no significant differences in depressive symptomatology between patients who used an app compared to those in the control group (25–27, 29–31). It is noteworthy that three of these six studies found within-group improvements in depressive symptomatology in both the app intervention and the control group. In the study by Hur et al. (25), there was a significant reduction in BDI-II scores within both the app intervention group and the control group (which also used an app, although only for recording mood state and sleep quantity and quality) after three weeks. Something similar was observed in the study conducted by Ly et al. (26), who found large within-group size effects in depressive symptomatology in both the app intervention group and control group (i.e., app based on mindfulness). In this same vein, Watts et al. (31) found significant differences within both groups (i.e., the app intervention group and the same intervention in a computer version) in depressive symptoms assessed by the PHQ-9 and the BDI-II. Finally, one outstanding result is that of the research conducted by Ly et al. (26), who found that the severity of initial depression could moderate the degree of response to the interventions. In patients with higher levels of depression (assessed using the PHQ-9), BA intervention was found to be superior to a mindfulness intervention; whereas in patients with lower levels of depression, the mindfulness-based intervention was found to be more effective than the BA intervention.

Regarding symptom severity, no significant improvement was shown in either of the two studies that measured it (30, 31). For its part, in the research of Tønning et al. (30) an improvement in the number of readmissions or in their duration was not shown.

Lastly, one of the three studies that analyzed the recovery rates found results favoring the efficacy of apps. Tønning et al. (30) found a significant improvement in recovery in the app intervention group compared to the control group. The study by Ly et al. (26) revealed significant recovery rates for both groups (BA intervention app and mindfulness intervention app), although there were no significant differences between them. Similarly, Ly et al. (27) found no differences between the app intervention group and control group in terms of recovery.

### Effectiveness on Quality of Life

No statistically significant differences between the app intervention group and control group were found in any of the five studies that assessed the quality of life of patients (25–27, 29, 30).

### Experiential Avoidance

In the two studies that assessed experiential avoidance, no significant differences between the app intervention group and control group were found (26, 27).

### Efficacy on Psychological Distress, Perceived Stress, and Anxious Symptoms

Regarding psychological distress, in the study by Watts et al. (31), it was found that patients who used an app on their smartphones significantly improved in this variable. However, in this study, no statistically significant differences were found when the app intervention group was compared to the control group. In relation to perceived stress, Raevuori et al. (29) found that the reduction in this variable in the intervention group was greater than that in the control group when measured at 20 and 32 weeks. Tønning et al. (30) found no differences in perceived stress between the app intervention group and control group. Finally, in the study by Hur et al. (25), a significant change in anxiety level was observed in the intervention group, compared to the control group, after a 3-week period of using the app. Nonetheless, no significant improvement in anxious symptomatology was found in any of the other three studies that assessed it (26, 27, 29).

### Efficacy on Other Outcome Variables

In a study using an intervention based on mindfulness, it was found that patients who used an app on their smartphones significantly improved both the internalization of mindfulness skills and their levels of resilience compared to the control group (29). Mantani et al. (28) observed that CBT participants experienced a lower burden of side effects in the total sample analysis, although this difference was not statistically significant compared to the control group. In the per-protocol sample analysis, the reduction in the burden of side effects in the app intervention was significant. Hur et al. (25) found a significant improvement in dysfunctional attitudes after a 3-week period of using the app in the intervention group, compared to the control group.

Finally, no statistically significant differences were found in self-esteem (25); sleep disturbances (29); empowerment (30); rumination (30); worrying (30); frequency, intensity, and burden of side effects [total sample, (28)]; psychosocial functioning (30); number of depressive episodes (30); wellbeing (30), satisfaction with care (30); adherence to medication (30); and BA (30) between the app intervention group and control group in the studies that included these variables.

## Discussion

The aim of this systematic review was to determine the efficacy of smartphone apps in patients with depressive disorders. After reviewing the seven articles included in this article, it can be concluded that the use of apps does not reduce depressive symptomatology nor improve the quality of life in patients diagnosed with depressive disorders when compared to an active control group. However, the effect of these interventions on perceived stress and anxious symptomatology remains unclear. The studies are characterized by a considerable degree of clinical and methodological heterogeneity, making it difficult to draw firm conclusions.

Regarding the efficacy of apps on depressive symptomatology, the analyzed studies show that app interventions do not reduce depressive symptoms in depressed patients when compared to an active control group. Only the study by Mantani et al. (28) found an improvement in depressive symptoms in a smartphone app intervention group, compared to a control group. However, in six of the seven studies of this review, no significant changes in depressive symptoms were observed in the app intervention group when compared to an active control group. When interpreting this result, the considerable level of heterogeneity among the selected studies should be taken into account. Different methodologies were used in the selected studies (including the application of different interventions, the combination of their components without verifying which ones were responsible for their efficacy, and the different measurements used at different times of the intervention and at different follow-up periods), and they include participants with different clinical characteristics (e.g., clinical severity). Furthermore, as noted by Kerst et al. (32), most studies include some sort of clinical support—although the vast majority is minimal, which makes it difficult to verify the unique effect of app interventions.

The findings of this review contrast with those of Hrynyschyn and Dockweiler (16), who found contradictory results on the efficacy of CBT-based apps in reducing depressive symptomatology in patients with mild or moderate depression, and highlight the considerable degree of heterogeneity of the articles included in their work. The review of these authors only included studies from 2015 onward, which only analyzed the efficacy of CBT-based apps in patients with mild to moderate depression (who in the majority of studies were recruited from a cutoff score on a depressive symptom questionnaire). Unlike the work of Hrynyschyn and Dockweiler (16), in this review, we consider studies published over a longer period of time, in which patients with a depressive disorder (diagnosed by a health professional) took part and received different types of treatment (including pharmacological treatment).

The results of this systematic review are not consistent with those of Serrano-Ripoll et al. (17), who found that app-based interventions resulted in a moderate reduction in depressive symptoms. These authors included in their meta-analysis participants with depressive symptoms, which in some studies had not necessarily been diagnosed by a health professional. In terms of interpreting the findings of Serrano-Ripoll (17), we should be taking into account that these authors analyzed studies comparing an app intervention group to a non-active control group, which could account for the effect found. App interventions rarely outperform active interventions [i.e., therapeutic interventions acting as an active control group; (6, 33)]. In our systematic review, we only included studies comparing an app intervention group to an active control group, which could make it difficult to identify any significant differences between the interventions. In fact, there are studies included in our systematic review that incorporate apps in both the intervention and control group, and although they do not find differences between the conditions, within-group improvements in both the intervention group and the control group in depressive symptoms (25, 26) are observed. Watts et al. (31) also found significant within-group improvement in the app intervention in relation to depressive symptoms (though they found no differences between the app and the same intervention in a computer-based version). The fact that within-group improvements in app intervention groups have been observed offers promise for future treatments. Another difference between our systematic review and that of Serrano-Ripoll et al. (17) is that our work only involves patients with depressive disorders diagnosed by a health professional. This allows us to ensure that the sample of patients between the different studies is as homogeneous as possible.

Regarding the effectiveness of the apps on quality of life, this variable did not improve significantly in any of the five studies that measured it. Therefore, the results of our review suggest that the app intervention does not appear to improve the quality of life in depressed patients. However, these results are tentative and should be regarded with caution. Hrynyschyn and Dockweiler (16) outline that the efficacy of app interventions in improving quality of life is inconclusive. We, however, state that it is possible that longer follow-up periods are required to detect improvements in the quality of life of depressive patients who are undergoing an app intervention. In this way, patients could apply the skills learned and achieve changes in their daily life.

With regard to the efficacy of apps for reducing perceived stress and anxiety, the results are unclear. A reduction in perceived stress was only significant in the study by Raevuori et al. (29), whereas Tønning et al. (30) found no significant improvement in perceived stress. Thus, no conclusions can be established. Additionally, a significant improvement in the reduction of anxious symptoms was found in only one (25) of the four studies that analyzed this variable. This is not consistent with the efficacy of apps to reduce anxiety in meta-analytic studies (15). As is the case with depressive symptomatology, it may be difficult to detect significant improvements in anxiety when an app intervention group is compared with an active control group. Finally, our results are in line with the findings of the review by Hrynyschyn and Dockweiler (16), who found contradictory results regarding the efficacy of apps to reduce anxiety in depressive patients. Therefore, the results regarding the efficacy of apps in reducing anxious symptoms in depressive patients are inconsistent.

In relation to experiential avoidance, in two of the seven studies that measured this variable, no significant differences between the app intervention group and control group were found. To the best of our knowledge, this variable has not been extensively explored in studies that employ apps to treat depression. Taking into account the rise of third-generation therapies (34), it is possible that in the coming years more studies will consider this variable.

With regard to other outcome variables, the studies analyze very different variables, which makes it difficult to draw firm conclusions. However, it is worth highlighting that in the study by Mantani et al. (28), in their per-protocol sample, there was a lower burden of side effects in the intervention group than in the control group at nine weeks. Although this was only analyzed in one study, it opens the possibility of combining apps with pharmacological treatment. The combination of treatments (i.e., app + medication) could be suitable for patients who do not respond to antidepressant treatment or who experience significant side effects while taking medication (28).

This systematic review has the following limitations. First, the selected articles showed a considerable degree of clinical and methodological heterogeneity, which explains the disparity in the results and makes it difficult to draw any firm conclusions. For example, the selected studies use different treatments; include various components (e.g., mindfulness, BA) without knowing which ones are responsible for the efficacy; use different measurement instruments (at different time points), different app interventions, and different control conditions; and include depressed patients with varying levels of clinical severity. This heterogeneity has also been found in studies that analyze the efficacy of app interventions (16, 17). In this review, we attempted to reduce heterogeneity by selecting studies conducted in clinical patients with depression diagnosed by a health professional. Second, although most studies use the PHQ-9 and the BDI-II to assess depressive symptomatology, other authors use different instruments, which makes it difficult to analyze the results regarding the main variable of interest. Third, only English-language studies were considered; therefore, language bias could not be ruled out.

The costs in social and public health policies associated with depression are high in all countries and continue to grow (4), and apps are accessible tools for most citizens. Despite the findings of our systematic review, we consider that the potential beneficial effect of apps merits investigation in future studies since they represent a low-cost and potentially far-reaching intervention of interest in the public health system. If they are shown to be effective, they could represent an opportunity for the prevention and treatment of depressive disorders. To date, however, the increased use of these interventions in clinical practice has not been accompanied by an increase in any associated scientific evidence (35), which generates a certain degree of mistrust on the part of users and professionals when it comes to recommending such interventions. In the future, it will be necessary to continue looking into the efficacy of apps for depressive patients and explore the mechanisms of change and moderators of the efficacy of the intervention (e.g., gender, age, socioeconomic and educational levels, symptom severity, medication, therapist-guided or unguided). Studies that aimed to address this question have not been able to identify moderating variables (6). To determine the mediators of change, it will be necessary to carry out dismantling studies that consider the components of the treatment in isolation (alone and/or in combination). Another step will be to analyze the efficacy of apps based on different effective treatments (e.g., psychotherapy and medication) and the role that apps play in changing the pharmacological treatment received by patients. Taking into account that intervention *via* apps is often applied to patients with subclinical depressive symptoms, it will be necessary to look into the efficacy of these interventions not only at an intervention level but also at a prevention level. Given the considerable degree of heterogeneity observed in the studies, it will be necessary to refine clinical trial methodology in the coming years.

On the other hand, in regard to applying apps in the clinical setting, some barriers have been found, such as a lack of engagement, which could explain the high dropout rates in some studies (36). Finding strategies that improve adherence to these interventions—such as offering feedback by a professional, daily monitoring of mood, or applying the principles of behavioral economics (15)—is a pending issue (36). In addition, the protection of personal data is a widespread problem when using apps to treat depressive disorders (14, 37) and is an important issue to consider. Multidisciplinary teams (e.g., psychologists, psychiatrists, computer scientists) should work on this issue. Lastly, the efficacy of using apps as a complementary intervention to the usual pharmacological and/or psychological treatment should be analyzed. It is possible that the inclusion of apps in conventional treatments could improve adherence to them (38).

According to the results of this systematic review, app interventions do not appear to reduce depressive symptomatology in depressed patients. However, in recent years, there has been an increase in their use both in the clinical setting and by mobile phone users (21, 23). Despite the potential beneficial effects of apps, we should bear in mind that apps are a double-sided coin, which also carries potential risks such as privacy and security concerns, and adverse effects of psychotherapy, among others (39). These adverse consequences could be minimized if the health professional keeps in mind the risk-benefit ratio of app interventions (39) and only recommends apps incorporating evidence-based interventions (40). When evidence-based apps are used in the clinical setting, health professionals should monitor their use by depressed patients to prevent them from taking risks, especially in the case of severely depressed patients. In depressed patients at risk of suicide, the use of an app could be of interest for suicide prevention (41) as well as to facilitate professional contact in emergency situations. However, the use of apps in patients at risk of suicide should always be complementary to usual treatment, and should support, but not replace, professional judgment (42). Lastly, we must not forget that, to date, app interventions cannot be regarded as a single treatment or be indiscriminately recommended.

Based on the results of this systematic review, it is concluded that app interventions do not appear to reduce depressive symptomatology nor improve the quality of life in depressive patients. However, the effect of these interventions on perceived stress and anxious symptomatology remains unclear. Additionally, the studies are characterized by a considerable degree of clinical and methodological heterogeneity, which makes it difficult to draw solid conclusions and points toward the need to improve the methodology of any future clinical trials.

## Data Availability Statement

Any inquiries can be directed to the corresponding author.

## Author Contributions

AH-G, MJV-F, and NA-G conceived the study, participated in its design, and coordination. AH-G and MJV-F carried out the literature searching and data extraction. All authors were involved in the interpretation of the data, drafting the manuscript, and revising it critically for important intellectual content. All authors contributed to manuscript revision, read, and approved the submitted version.

## Conflict of Interest

GL has been a consultant to or has received honoraria or grants from Janssen-Cilag, Otsuka-Lundbeck, Lilly, Astra-Zeneca, Angelini, CIBERSAM, and Instituto de Salud Carlos III. The remaining authors declare that the research was conducted in the absence of any commercial or financial relationships that could be construed as a potential conflict of interest.

## Publisher's Note

All claims expressed in this article are solely those of the authors and do not necessarily represent those of their affiliated organizations, or those of the publisher, the editors and the reviewers. Any product that may be evaluated in this article, or claim that may be made by its manufacturer, is not guaranteed or endorsed by the publisher.

## Footnotes

^1^Studies that included an active control group (i.e., groups that employed an alternative psychological and/or pharmacological intervention) were selected.

^2^In all studies, patients received a professional diagnosis of depressive disorder. The diagnostic criteria used in each study as well as the diagnostic method employed can be found in Table 1.

^3^Depressive symptomatology was the main outcome variable in all studies, except in the study by Tønning et al. (30), in which it was included as a secondary variable. Ly et al. (27) included depressive symptoms as assessed using the BDI-II as a primary outcome measure and depressive symptoms as assessed using the PHQ-9 as a secondary outcome measure.
